# Traumatic bilateral acetabular fracture secondary to high-energy trauma in healthy adults

**DOI:** 10.1186/s12893-023-02302-1

**Published:** 2024-01-03

**Authors:** Guy Romeo Kenmegne, Chang Zou, Yixiang Lin, Yijie Yin, Shenbo Huang, Yue Fang

**Affiliations:** 1 Department of Orthopedics, West China Hospital of Sichuan University, Chengdu, 610041 China; 2 Trauma center, West China Hospital of Sichuan University, Chengdu, 610041 China

**Keywords:** Bilateral acetabular fracture, Healthy adults, High energy trauma, Road traffic accidents, Open reduction and internal fixation, Fracture fixation

## Abstract

**Background:**

Bilateral acetabular fracture is a very rare presentation among the trauma patients, as the pattern and the degree of the forces required to fracture both acetabula is very unique. The primary purpose of this study is to report a series of adult patients presenting with post-traumatic bilateral acetabular fracture without any history of pathological or metabolic bone disease.

**Patients and methods:**

In this retrospective study, 18 cases of traumatic bilateral acetabular fracture were included. There was predominance of both column (four patients on left and six on right) followed by anterior column (two patients left and four on right) and posterior wall (three patients left and right). They were treated surgically through open reduction and internal fixation. All cases were followed up for at least 13 months. Matta’s criteria were used for radiological evaluation on plain radiographs. Functional outcome was evaluated using the Merle d’Aubigne and postel score at final follow-up.

**Results:**

No patients were lost during the follow-up period; there was one case of surgical site infection. There were three cases of postoperative osteoarthritis, one case of heterotrophic ossification, one case of persistent sciatic nerve palsy and one case of lateral femoral cutaneous nerve palsy. The radiological evaluation according to Matta’s criteria revealed anatomic reduction in 12 patients, imperfect reduction in three patients while other three patients had poor reduction. According to modified Merle d’Aubigne and Postel score, 10 cases were rated as excellent, five cases as good and three cases presented fair (one case) to poor (two cases) results.

**Conclusion:**

We report an unusual case series of bilateral acetabular fracture successfully managed surgically with good clinical outcome. With the increasing incidence of route traffic accidents, such cases would probably be recurrent in the upcoming years.

## Introduction

Injuries to the acetabula characterized by Bilateral fracture of both acetabula resulting from high energy trauma has already been reported in the literature following traffic accidents in adult population [1]; however these complex injuries remain extremely uncommon in trauma patients [2]. Several previous scholars have reported similar injuries occurring in seizure patients as a result of seizure crisis [3–7]; there were few cases reported following low energy trauma in elderly patients with history acetabular insufficiency [8, 9] These special types of fractures are extremely complex and the relevant management algorithm are extremely challenging among trauma surgeons [10]; most trauma surgeons have less experience in these injuries, as a results most patients may not appropriate medical and surgical care; additionally, as it can be associated to hip dislocation and/or other vital organs injury even death if excessive blood loss is involved [1, 11], these patients usually require multidisciplinary medical care. To the best of our knowledge, there are no previous important literatures reporting traumatic bilateral acetabular fracture in a patient series. Therefore, the primary aim of this study was to report a series of adult patients presenting with post-traumatic bilateral acetabular fracture without any history of pathological or metabolic bone disease.

### Patients and methods

This monocentric retrospective study was carried out in a level one trauma center and considered all patients treated consecutively from January 2014 to May 2022 admitted to our hospital with a diagnosis of traumatic bilateral acetabular fracture. Eighteen patients with bilateral acetabular fracture treated surgically were included.

All procedures were done by the same medical team and coauthors of this study. The inform consent about the surgical technique and its possible related complications was signed by the patients. They should be aware that acetabular fracture patients are exposed to massive blood loss which can be potentially life-threatening for the patients. Additionally, they should be informed of the fact that, as most of them present in the poly-trauma condition, they are prone major complications such as wound complications, hemodynamic shock, DVT/PE and nerve palsies. The study was approved by the local Ethics Committee of our institution.

### Inclusion criteria

We included all patients with all types of Judet-Letournel acetabular fracture requiring internal fixation and all patients who were managed surgically.

### Exclusion criteria

1. Patients with bone metabolic disorder (pathological fracture).
2. Patients managed conservatively.
3. Patients with less than 1 year follow-up.

## Methods

All patients were admitted at the emergency department. The advanced trauma life support guidelines were followed in the resuscitation protocol. Once the patients were hemodynamically stable, routine plain radiographs of the pelvis (anteroposterior view, obturator view, iliac view inlet view and outlet view) and 3D CT scan were performed. If on plain radiograph, acetabular fracture was diagnosed with posterior wall combined with femoral head posterior dislocation, closed reduction was done under anesthesia and skeletal traction was applied thereafter.

### Surgical techniques

In the combined S + IF approach, our patients were placed in supine position. After preliminary skin preparation and aseptic protocols; the Stoppa incision started at two finger-breadths (1–2 cm) above the pubic symphysis. The incision continued deep to the rectus abdominis which was retracted, the urinary bladder protected with gauze; the exposure continued deeply with lateral retraction of neurovascular bundle, an attention should be paid on the corona mortis, which is immediately ligated if encountered. The true pelvis rim along with the quadrilateral plate as well as the posterior column is usually exposed from front to back after dissection of ilio-pubic and obturator fascia.

The IF incision started at half distance of the iliac crest to the ASIS, identification and isolation of the lateral femoral cutaneous nerve; followed by a blunt separation of the iliac and iliopsoas muscles, exploration, release and protection of the femoral vessels. The reduction strategy includes the combination of ball-spike pusher, used to provide an outwards pressure forces on the displaced fragment, supported by the Hohmann lever inserted into the greater sciatic notch to support the posterior column, all assisted by the traction of the ipsilateral limb to ease the reduction. In case of central dislocation, the use of Shantz nail is usually required to pull the femoral head laterally in order to ease the reduction.

When the K-L approach needs to be added, the patient has to be placed in prone position. Postero-laterally, a 15–20 cm K-L incision is applied; followed by a blunt separation of the gluteal muscles, exploration and release the sciatic nerve in case of any local compression. In this approach, the reduction technique consists of the use of ball-spiked pusher to apply an inward pressure on the fractured part of the posterior wall. Because most fractures involving the posterior wall often present with posterior dislocation of the affected hip joint, a traction on the affected leg simultaneously with flexion and extension of the knee and hip respectively is usually needed during the operation to facilitate the reduction process.

Irrespective of the aforementioned operative approaches used, in all cases, the fixation steps consist of temporary fixation, intraoperative C-arm fluoroscopy to appreciate the quality of the reduction; finally, a pre-bent plate and screws can be used to permanently fix the fracture.

### Perioperative management

Routinely, Patients received second-generation cephalosporin 30 min preoperatively and 24 h postoperatively to prevent infection. All patients received 1-2 g of intravenous tranexamic acid prior the incision; the dose was repeated 3 hrs after the after the incision and Q6h for 24 hrs following the procedure. Patients with patients with concomitant limb fracture were planned for staged surgeries in respect to their physiological conditions. Postoperatively, patients were allowed to undergo isometric contraction exercises of the quadriceps of the femoris and active and passive flexion and extension training of the ankle joint to reduce oedema of the lower extremities. Low-molecular weight heparin (0.2–0.4 ml, according to the weight of patients) was routinely injected until discharge to prevent deep vein thrombosis. Oral rivaroxaban and loxoprofen sodium tablets were prescribed at 10 mg/day and 180 mg/day (three times a day) respectively for 3 weeks after discharge to prevent thrombosis and pain in a later stage. All patients were recommended 75-150 mg indomethacin three times a day to prevent the postoperative heterotrophic ossification. Partial weight-bearing and full weight-bearing exercises were gradually increased according to the rate of fracture healing on X-ray radiography. All patients were required to return to the outpatient clinic for follow-up at one, three, six, nine, and 12 months postoperatively and then once every 6 months thereafter.

### Outcome measurement

**Fig. 1:**
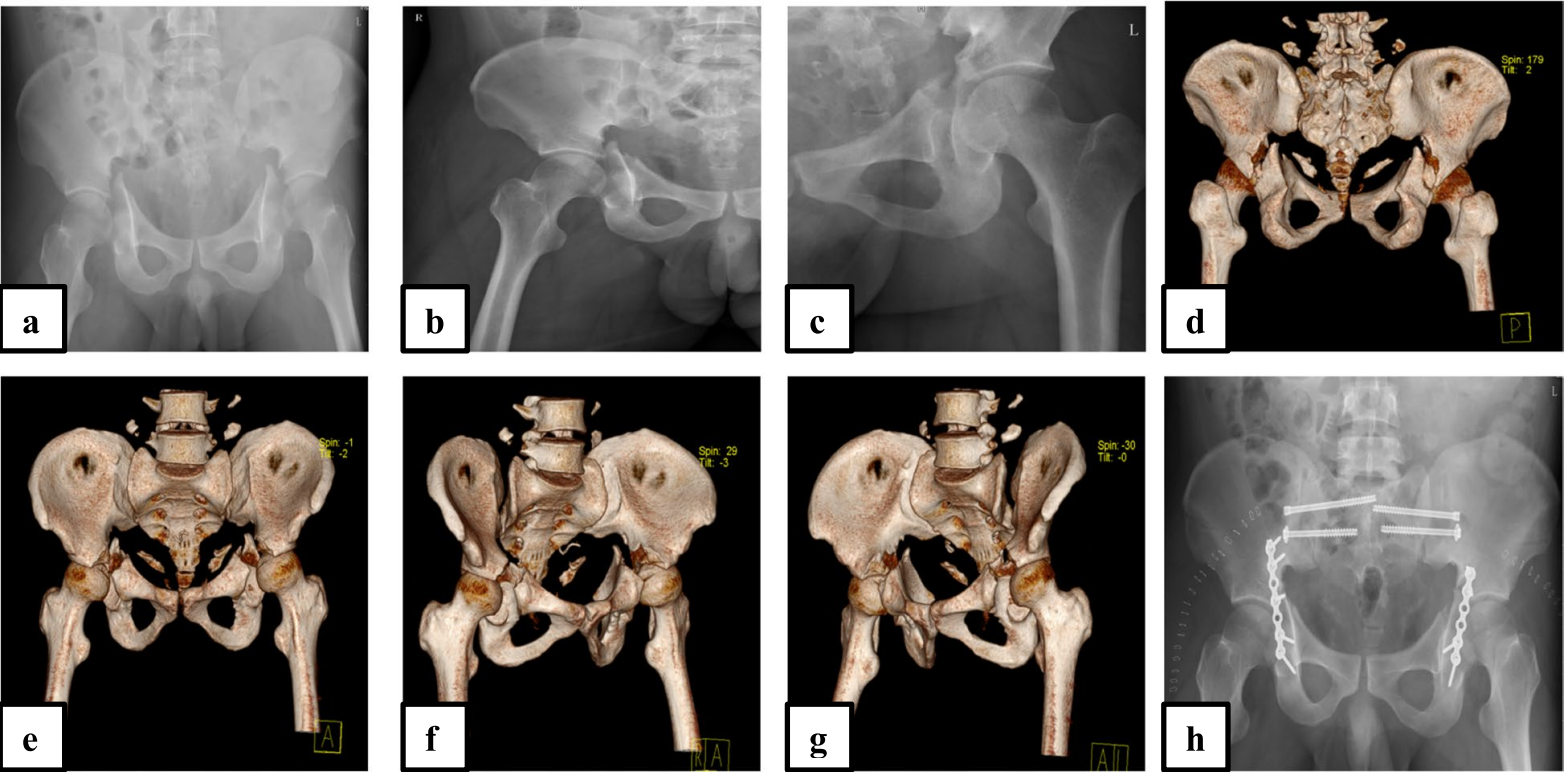
A 53 years-old male patient who sustained a bilateral acetabular fracture (Letournel-Judet transverse type) following route traffic accident: (**a**) preoperative plain radiograph anteroposterior view; (**b**) and (**c**) preoperative radiograph showing left and right acetabulum fracture; (**d**) 3D Ct scan image posterior view showing posterior displacement; (**e**) 3D Ct scan image anterior view showing evidence of both acetabula fracture, preoperative 3D Ct scan image showing Tile C type Pelvic fracture with bilateral sacroiliac joint dislocation (**f**) and (**g**), sacral fracture); (**h**) postoperative plain radiograph anteroposterior view

All clinical data for operative time, surgical blood loss, length of stay, postoperative complications; the estimated blood loss was evaluated using the standard clinical method where blood loss was taken as the volume recovered in drains plus the intraoperative blood loss. The total unit of allogeneic blood transfused was calculated from the resuscitation period at the emergency department (if any) to the patient’s discharge. Matta’s criteria of reduction to assess the accuracy of reduction and the presence of residual displacement on plain radiographs. It was graded as anatomic (when there was presence of 0-1 mm fracture gap), imperfect (2-3 mm fracture gap), and poor (if presence of > 3 mm fracture gap) according to the scoring system published by Matta [12]. Clinical outcomes were measured using the Matta modification of the Merle d’Aubigne score and the reports were expressed as Excellent, good or poor [13]. Early postoperative complications such as infection, neurovascular injuries, and hematoma were also recorded. The imaging assessment for postoperative osteoarthritis was evaluated following the Kellgren-Lawrence [14] Radiological assessment criteria of osteoarthritis. The Fig. 1 illustrates a typical case of bilateral acetabular fracture patient managed operatively.

### Statistical analysis

In this study, all statistical analyses were conducted using SPSS 26.0 (SPSS, IBM, USA); descriptive statistic was chosen as method of analysis, continuous variable were expressed as mean ± standard variation while numbers were described as percentage.

## Results

The patient was male in 11 cases, while seven patients were female. The mean age was 46 ± 13.7 SD years (ranging from 19 to 69 years). Mean follow-up 32.77 ± 11.21 months (range 13–48 months). The mode of trauma was route traffic accident (RTA) in 16 cases and fall from height in two patients. The mean injury severity score (ISS) was 26.61 ± 8.41 (range 12–41). On both side, there was predominance of both column (four patients on left and six on right) followed by anterior column (two patients left and three on right) and posterior wall (three patients left and right). All patients were operated within the 15 days (mean 9.27 ± 3.17); the mean hospital length of stay (LOS) was 28.33 ± 13.6 days. Radiographic evaluation according to Matta’s criteria revealed anatomic reduction achieved in 13 cases while there was imperfect to poor reduction in five cases (three imperfect and two poor).

According to the modified Merle d’Aubigne score, 10 cases were rated as excellent, six cases as good and three cases presented fair (one) to poor (one) result. There was one case of heterotrophic ossification requiring surgical management in one case (case five), one case of sciatic nerve palsy in a patient in whom the primary injury was associated with sciatic nerve entrapment and contusion which required intraoperative exploration and nerve release; the unique case of lateral femoral cutaneous nerve palsy was found in one patient and was termed as iatrogenic; during the hospitalization period, six cases of pneumonia was diagnosed with one case of acute respiratory response syndrome (ARDS), in late follow-up, 3/18(16.66%) were found with radiological signs of osteoarthritis (using Kellgren-Lawrence criteria); meanwhile only one out of three expressed typical symptoms of osteoarthritis and was classified as grade IV according to Kellgren-Lawrence classification system. Additionally, one patient presented with persistent lateral femoral cutaneous nerve palsy (Table 1).

**Table 1:** Clinical data of patients

case	age	sex	MOI	ISS	Fracture classification		Surgical approach		Matta criteria	Merle d’Aubigne score	TTS	LOS	Follow-up (months)	complications
					L	R	L	R						
1	59	F	RTA	41	Ts	AW	IF	S + IF	Anatomic	Good	14	30	45	-pneumonia
2	19	M	RTA	25	BC	PW	S + IF	K-L	Imperfect	Excellent	14	19	40	-pneumonia
3	53	M	RTA	12	Tr	Tr	K-L	K-L	Anatomic	good	9	20	48	-osteoarthritis
4	25	M	RTA	25	PW	BC	K-L	S + IF	Anatomic	Excellent	7	16	30	-pneumonia
5	48	M	FFH	28	Ts + PW	AW	S, IF+K-L	IF	Anatomic	Poor	13	63	36	-heterotrophic ossification
6	54	F	RTA	31	AW	BC	IF+S	S + IF	poor	Fair	5	42	25	-osteoarthritis, pneumonia
7	52	M	RTA	22	AC + PSTr	AC	S, IF+K-L	S + IF	Anatomic	Good	7	34	36	-Sciatic nerve palsy
8	55	M	RTA	27	PW	BC	KL	S + IF	Anatomic	Excellent	6	18	24	–
9	43	F	RTA	41	AW	BC	S + IF	S + IF	imperfect	Excellent	9	16	45	-pneumonia
10	41	M	RTA	38	Tr + PW	BC	S, IF+K-L	S + IF	Anatomic	Excellent	8	15	38	Lateral femoral cutaneous nerve palsy
11	23	F	FFH	22	AC	Ts	S + IF	S + IF	poor	Good	15	43	40	-osteoathritis
12	50	M	RTA	22	PW	AC	K-L	S + IF	imperfect	Excellent	6	31	23	–
13	69	M	RTA	24	AC + PSTr	PC	S,IF	K-L	Anatomic	good	8	33	48	-pneumonia -ARDS
14	47	F	RTA	13	AC	BC	S + IF	S + IF	Anatomic	Excellent	11	25	19	–
15	30	M	RTA	24	BC	PW	S + IF	K-L	Anatomic	Good	10	23	39	-wound complication
16	61	M	RTA	34	BC	AC	S + IF	S + IF	Anatomic	Excellent	6	10	26	–
17	48	F	RTA	32	BC	Tr + PW	S + IF	S,IF+K-L	Anatomic	Excellent	7	25	13	–
18	51	F	RTA	18	Ts	PC + PW	S + IF	K-L	Anatomic	Excellent	12	47	15	–

*M* male: *F* female: *L* left: *R* right: *ISS* injury severity score: *MOI* mechanism of injury: *FFH* falling from height: *RTA* route traffic accident: *BC* both column: *Ts* T-shape: *PW* posterior wall: *AC* anterior column: *PC* posterior column: *PW* posterior wall: *S* stoppa: *IF* iliac fossa: *Tr* transverse: *PST* posterior-semi transverse: *TTS* time to surgery(days): *LOS* length of hospital stay (days): *ARDS* acute respiratory distress syndrome

The mean operation time of 188.94 ± 38.74 min and the mean total estimated blood loss (intraoperative and drainage blood) was 751.88 ± 58.33 mL (range 630-850 mL) and the mean allogeneic unit of blood transfused was 3.88 ± 1.27 units (range 2–6 units) (Table 2).

**Table 2:** patients’ intraoperative data

Variables	Values
Operation duration (minutes)	188.94 ± 38.74
EBL(mL)	751.88 ± 58.33 mL
Total unit of blood transfused (units)	3.88 ± 1.27
EBL = estimated blood loss.	

All our patients presented some additional injuries related to the nature of the injuries. There were 10 injuries of trauma to the head and neck region distributed as traumatic brain injury (TBI) in six patients, maxillofacial trauma in three cases and eye trauma in one case; 12 conditions of chest trauma(among which four cases had ribs fracture and eight cases had simple lung contusion). There were five conditions of abdominal trauma, one case of visceral organ ruptures and four cases of simple organ contusion. We had three patients with lumbar vertebral body fractures (in two cases, the mechanism of injury was fall from height). In all cases, there were involvement pelvic ring fractures, 15 patients presented additional lower limb fractures and eight cases of fractures in the upper limb (Table 3).

**Table 3:** Regional distribution of concomitants lesions

Regions	Numbers of lesions	Clinical lesions	
Head and neck	10	Traumatic brain injury	6
		Maxillo-facial trauma	3
		Ocular trauma	1
Chest	12	Ribs fracture	4
		Lung contusions	8
Abdomen	5	Visceral organ rupture	1
		Visceral organ contusion	4
spine	3	Vertebral body fracture	3
pelvis	21	Pelvic ring fracture	18
		Pelvic organ lesions	3
Upper limb	9	fractures	8
		Soft tissue contusion	1
Lower limb	17	Fractures	15
		Soft tissue contusion	2

**Fig. 2:**
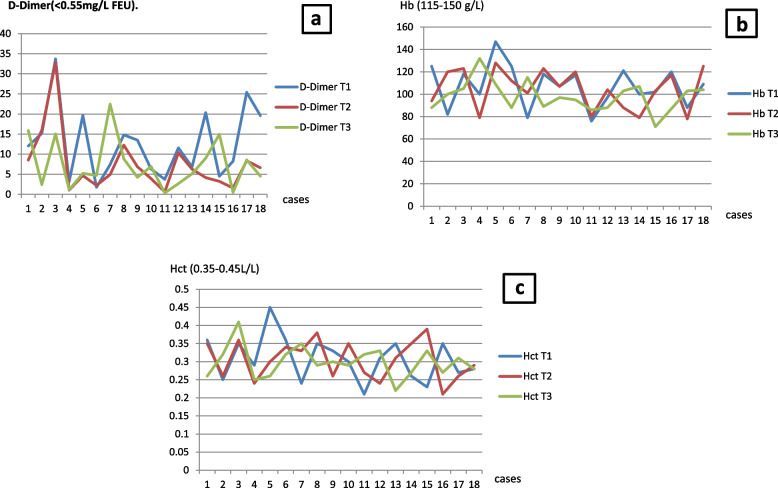
Perioperative blood laboratory investigation: **A** = perioperative D-Dimer; **B** = perioperative hemoglobin; **C** = perioperative hematocrit; (T1 = admission day; T2 = 1 day pre-operation; T3 = first day post-operation)

The laboratory screening of D-Dimer regularly yielded a decreased level from the admission to the operation day and even postoperatively however, the values remained above the normal limit (< 0.55 mg/L FEU). The hemoglobin and hematocrit level in our patients is generally relatively low around the perioperative time, indicating the need of blood group and rhesus matching for any eventual need of blood transfusion. Cell saver machine was used intraoperatively in all our patients. **(**Fig. 2**).**

## Discussion

Bilateral acetabular fracture is a very rare presentation among the trauma patients, with the nature of the forces required to fracture both acetabula being very unique [2]. Previous scholars have reported such cases in patients secondary to seizure crisis, pregnant woman with osteoporosis condition and elderly individual with low bone density [1, 3–5, 7–9, 15–17]. Few cases including patients with previous underlying medical condition such as renal insufficiency, history of steroid and radiotherapy were also encountered within the literature [3, 8]. Although most studies described fracture of both acetabula, mostly during seizure crisis, and after low energy trauma in elderly with history of osteoporosis, one case have similarly been reported after low-energy trauma in a younger (15-year-old) male patient; with the immaturity of bones being incriminated as the most important underlying risk factor [18]. In this study, we are reporting a case series of bilateral acetabular fracture in young healthy individuals secondary to high energy trauma, without history of underlying comorbidity such as bone degenerative disease, history of antiepileptic, corticosteroid or any other medications.

The challenges found in bilateral acetabular fracture reside in both their clinical presentation and the complexity of their management; because of the vulnerability of the bowel or urogenital systems, there is an increased risk of contamination surgical site infection and sepsis [19]. Such patients often present with important comorbidities such as traumatic brain injury (TBI), rib fractures, lung contusions, and mesenteric irritation with respiratory distress and infection [20]. In the current study, six cases (among which five were all admitted in ICU with endotracheal intubation) were complicated by pneumonia; patients with chest trauma were all diagnosed with pleural effusion on CT scan, however, only one patient required drainage by pleural tap.

The study observed one cases of wound complication (wound infection) in a young adult obese patient (BMI = 33) with unknown exact underlying cause, however, bedside debridement, antibiotic therapy and frequent wound dressing lead to healing without further complications.

Additionally, all patients presented at least one or two concomitants injuries: TBI, chest trauma (lung contusion and ribs fractures), internal organ injury or appendicular skeleton fracture; moreover, two patients presented clinically with sciatic nerve injury symptoms, nevertheless, the intraoperative exploration and release of sciatic nerve compression, followed with postoperative oral mecobalamin 500 mcg TID for three to 4 weeks, completely resolved symptoms in one patient while minor residual symptoms remained persistent in the second patient (patient number seven, Table 1).

In Most cases of acetabular fracture following high-velocity trauma, posterior hip dislocation is usually simultaneously described especially when posterior wall/column of acetabulum is involved; these require emergency reduction [21]. In the current study, there was posterior hip subluxation in four patients with sciatic nerve entrapment in two cases. The cases of sciatic nerve entrapment were released intra-operatively. According to the literature, sciatic nerve palsy is a common complication following acetabular fracture, particularly posterior wall fracture or when the fracture is associated to hip dislocation [22]. In our clinical experience, almost all acetabular fractures involving the posterior wall or posterior column are accompanied with posterior hip dislocation sometimes requiring emergency manual or surgical reduction; additionally these patients usually present symptoms of sciatic nerve contusion injury. The intraoperative exploration/ release accompanied with postoperative oral mecobalamin are usually conducive to good results and patients often recover from their symptoms without sequel.

The management algorithm of patients with fracture of both acetabula is still controversial among specialists; some authors have reported good clinical outcome with conservative management [3, 5]; while others have recorded cementless THA as management plan [6, 7, 9, 15, 16]; in contrast, other scholars have regarded ORIF as the gold standard approach [8, 15]. However percutaneous fixation has also been been suggested, but this can only be applied in cases with non-displaced or fairly displaced fracture [23]. In our study, we considered surgical management (ORIF) in all our patients. The approaches included the modified Stoppa aiming to get access to both sides and fix the inner wall of each acetabulum, combining with bilateral iliac fossa approaches, principally to address the roof of the acetabulum and the iliac crest fracture; additionally, a posterior K-L approach was considered if posterior wall was involved.

In terms of operation steps, it is recommended to start reduction from the periphery to the center of the acetabulum, the larger fracture portion being addressed before attempting to reduce the smaller in other to achieve adequate fracture reduction and perfect articular surface reconstruction. However, given the complexity of the fracture, the choice of the above steps were often influenced by the degree of fracture displacement and the hip joint subluxation limiting the reduction; therefore, we usually prefer to start by addressing the hip subluxation, following with the reduction of displaced fractured fragments and finally we proceed a definitive and stable fixation with plate and screw.

In late follow-up, 16.66% of patients presented with osteoarthritis, diagnosed on plain radiographic image, moreover, the analysis of the postoperative radiographs revealed that all three patients had poor fracture reduction according to Matta’s criteria; we came to an agreement that this mal-reduction could be the primary cause of the osteoarthritis cases found. According to some previous authors, posttraumatic arthritis (PTA) is a potential chronic complication after acetabular reconstruction regardless the operative approach (either ORIF or THA), and cannot only be related to postoperative joint incongruity, but also the damage to the articular cartilage caused by trauma itself; therefore, achieving perfect anatomical reduction is the most important factor to predict good functional outcome [24]. Several authors supported that, early surgical procedure, ideally within 7 days (or in less than 2 weeks) when ORIF is considered and good anatomical reduction of the articular surface are cornerstone for prevention of PTA [25].

However, because respecting the required timing of the operation in patients with acetabular fracture is quite challenging due to the poly-trauma state in which they usually arrive (ISS > 16), they often require a certain resuscitation protocol and stabilization to fulfill the biological and physiological conditions for operations; additionally, most of these patients (as in our study 6/18) may require admission in ICU for active resuscitation; these patients, usually under respiratory support, develop pneumonia which in contribution with all the above mentioned factors can further delay their operation.

The current study reported one case of postoperative heterotrophic ossification, in a patient 15 months following operative fixation. Heterotrophic ossification has long been discussed as related to posterior Kocher-Langenbeck approach to acetabulum [26–28]. The available literature proved the efficacy of prophylactic use of nonsteroidal anti-inflammatory drug indomethacin at a dosage of 75 mg Q12h or 25 mg Q8h and radiotherapy in decreasing the risk of postoperative heterotrophic ossification [28–30]. It is our routine to prescribe indomethacin to all our pelvic and acetabular fracture patients regardless the operative approach used during the internal fixation. In patients with traumatic brain injury there is 10 to 53% risk of developing heterotrophic ossification in major joints in the body; in these patients, the literature support the use of bisphosphonate rather than Indomethacin [29]. The case of heterotrophic ossification described in this study had concomitant TBI and was prescribed indomethacin instead of bisphosphonate, it is therefore unclear if this condition was related to the initial injury, the operative approach or the prevention protocol used.

In the current series, there was a persistence of lateral femoral cutaneous nerve palsy (due to iatrogenic lesion) in one of our patient. According to the literature, there is high vulnerability of lateral femoral cutaneous nerve due to its anatomical variations exposing it to intraoperative iatrogenic injuries [28, 31]. A blunt dissection of soft tissue over the iliac crest periosteum during the incision may help to identify the lateral femoral cutaneous nerve however regardless its location and course and significantly reduce the risk of its iatrogenic of injury.

Bleeding is considered as one of the important factors which characterizes pelvic and acetabulum fractures; According to the literature, the estimated blood loss due to the initial injury and the operative procedure are considered to be as high as 1232–2818 mL [32]. To minimize the risk of excessive bleeding, we routinely used perioperative administration of tranexamic acid (TXA). In this study, the estimated total blood loss was 751.88 ± 58.33 mL (ranging from 630 to 850 mL). This result was consistent with other previous studies on the efficiency of intravenous TXA in reducing blood loss in pelvic and acetabular fracture patients [33].

Patients with an ISS of ≥16 have an increased risk factor of deep vein thrombosis (DVT), which risk is significantly increased when lower extremity fracture is involved [34]. In the current study, the mean ISS was 26 on admission, and an elevated D-dimer (mean = 12.64 on admission) exposed to a risk of DVT, indicating a need of chemotherapeutic anticoagulant prophylaxis.

Additionally in relation to the elevated ISS, it is an indicator of the severity of the injury and can be an influencing factor increasing the hospital length of stay. In our study, our data demonstrated that patients with elevated ISS had longer hospital LOS. Overall the mean hospital length of stay (LOS) was 28.33 ± 13.6 days in our series; this considered to be relatively long and regarded as an indicator of the severity of the initial trauma.

One of the limitations in this study is the lack of consistent studies available in literature to compare with our findings; secondly, the retrospective nature of the study makes it susceptible to biased; and finally, the limited numbers of patients enrolled in the study could not allow us to draw some strong conclusions with our findings.

## Conclusion

In conclusion, we report an unusual case series of bilateral acetabular fracture in healthy adult patients successfully managed with ORIF. To our knowledge, this is one of the rare case series of bilateral acetabular fracture due to high-energy trauma ever reported in the literature and constitute the main strength of this study. However, with the increasing incidence of RTAs, such cases would probably be recurrent in the upcoming years.

## Data Availability

The datasets used and/or analyzed during the current study are available from the corresponding author on reasonable request.

## References

[1] Nehme AH, Matta JF, Boughannam AG, Jabbour FC, Imad J, Moucharafieh R (2012). Literature review and clinical presentation of bilateral acetabular fractures secondary to seizure attacks. Case rep orthoped..

[2] Stevens JM, Shiels S, Whitehouse MR, Ward AJ, Chesser TJ, Acharya M (2020). Bilateral acetabular fractures: mechanism, fracture patterns and associated injuries. J Orthop..

[3] Granhed HP, Karladani A (1997). Bilateral acetabular fracture as a result of epileptic seizure: a report of two cases. Injury..

[4] Friedberg R, Buras J (2005). Bilateral acetabular fractures associated with a seizure: a case report. Ann Emerg Med..

[5] Takahashi Y, Ohnishi H, Oda K, Nakamura T (2007). Bilateral acetabular fractures secondary to a seizure attack caused by antibiotic medicine. J Orthop Sci..

[6] Heyer JH, Thakkar SC, Zittel K, Tozzi JE (2020). Bilateral acetabular fractures treated with delayed total hip arthroplasty. Arthroplasty today..

[7] Van Heest A, Vorlicky L, Thompson RC (1996). Bilateral central acetabular fracture dislocations secondary to sustained myoclonus. Clin Orthop Relat Res..

[8] Benz D, Lim P, Balogh ZJ (2019). Acute atraumatic bilateral acetabular insufficiency fractures. J Orthop Surg (Hong Kong)..

[9] Rosa MA, Maccauro G, D'Arienzo M (1999). Bilateral acetabular fracture without trauma. Int Orthop..

[10] Ingle M, Bhalotia A, Chandele V (2018). Bilateral anterior column acetabulum fracture following road traffic accident: a rare presentation. J orthopaed case rep..

[11] Qin W, Fang Y (2021). Traumatic asymmetrical bilateral hip dislocation: a rare case report. Joint diseas relat surg..

[12] Matta JM (1996). Fractures of the acetabulum: accuracy of reduction and clinical results in patients managed operatively within three weeks after the injury. J bone joint surg Am..

[13] Matta JM, Mehne DK, Roffi R (1986). Fractures of the acetabulum. Early results of a prospective study. Clin Orthop Relat Res..

[14] Kellgren JH, Lawrence JS (1957). Radiological assessment of osteo-arthrosis. Ann Rheum Dis..

[15] Aynaci O, Kerimoglu S, Ozturk C, Saracoglu M (2008). Bilateral non-traumatic acetabular and femoral neck fractures due to pregnancy-associated osteoporosis. Arch Orthop Trauma Surg..

[16] Tempelaere C, Diviné P, Bégué T (2019). Early simultaneous bilateral total hip arthroplasty for the management of bilateral acetabular fracture in an elderly patient. Arthroplasty today..

[17] Balcarek P, Dresing K, Walde TA, Tezval M, Stürmer KM (2009). Myoclonus-induced bilateral acetabular fracture dislocations. J Arthroplast..

[18] Nodzo SR, Hohman DW, Galpin RD (2012). Bilateral acetabular fractures in an adolescent after low-energy trauma. Pediatr Emerg Care..

[19] Scheinfeld MH, Dym AA, Spektor M, Avery LL, Dym RJ, Amanatullah DF (2015). Acetabular fractures: what radiologists should know and how 3D CT can aid classification. Radiograph..

[20] Bakhshayesh P, Weidenhielm L, Enocson A (2018). Factors affecting mortality and reoperations in high-energy pelvic fractures. Eur J Orthop Surg Traumatol..

[21] Sahin O, Ozturk C, Dereboy F, Karaeminogullari O (2007). Asymmetrical bilateral traumatic hip dislocation in an adult with bilateral acetabular fracture. Arch Orthop Trauma Surg..

[22] Jindal K, Aggarwal S, Kumar P, Kumar V (2019). Complications in patients of acetabular fractures and the factors affecting the quality of reduction in surgically treated cases. J clin orthopaed trauma..

[23] Caviglia H, Mejail A, Landro ME, Vatani N (2018). Percutaneous fixation of acetabular fractures. EFORT open rev..

[24] Fakru NH, Faisham WI, Hadizie D, Yahaya S (2021). Functional outcome of surgical stabilisation of acetabular fractures. Malaysian orthopaed j..

[25] Cahueque M, Martínez M, Cobar A, Bregni M (2017). Early reduction of acetabular fractures decreases the risk of post-traumatic hip osteoarthritis?. J clin orthopaed trauma..

[26] Manzoor QW, Sultan A, Mir BA (2021). Osteosynthesis of common acetabular fractures operated on through a single posterior (Kocher-Langenbeck) approach with or without trochanteric Flip osteotomy. A case series. Ortopedia traumatol rehab..

[27] Elson RA, Letournel E, Judet R (2012). Fractures of the acetabulum.

[28] Kelly J, Ladurner A, Rickman M (2020). Surgical management of acetabular fractures - a contemporary literature review. Injury.

[29] Ranganathan K, Loder S, Agarwal S, Wong VW, Forsberg J, Davis TA, Wang S, James AW, Levi B (2015). Heterotopic ossification: basic-science principles and clinical correlates. J bone joint surg am..

[30] Baschera D, Rad H, Collopy D, Zellweger R (2015). Incidence and clinical relevance of heterotopic ossification after internal fixation of acetabular fractures: retrospective cohort and case control study. J Orthop Surg Res..

[31] den Brave PS, Vas Nunes SE, Bronkhorst MW (2015). Anatomical variations of the lateral femoral cutaneous nerve and iatrogenic injury after autologous bone grafting from the iliac crest. J Orthop Trauma..

[32] Raobaikady R, Redman J, Ball JA, Maloney G, Grounds RM (2005). Use of activated recombinant coagulation factor VII in patients undergoing reconstruction surgery for traumatic fracture of pelvis or pelvis and acetabulum: a double-blind, randomized, placebo-controlled trial. Br J Anaesth..

[33] Gümüştaş SA, Çelen ZE, Onay T, Abul MS, Çevik HB (2022). The efficiency and safety of intravenous tranexamic acid administration in open reduction and internal fixation of pelvic and acetabular fractures. Europ J Trauma Emergency Surg..

[34] Magnussen RA, Tressler MA, Obremskey WT, Kregor PJ (2007). Predicting blood loss in isolated pelvic and acetabular high-energy trauma. J Orthop Trauma..

